# Crystal Structure of Human Myotubularin-Related Protein 1 Provides Insight into the Structural Basis of Substrate Specificity

**DOI:** 10.1371/journal.pone.0152611

**Published:** 2016-03-28

**Authors:** Seoung Min Bong, Kka-bi Son, Seung-Won Yang, Jae-Won Park, Jea-Won Cho, Kyung-Tae Kim, Hackyoung Kim, Seung Jun Kim, Young Jun Kim, Byung Il Lee

**Affiliations:** 1 Research institute, National Cancer Center, Goyang, Gyeonggi 10408, Republic of Korea; 2 Department of Biomedical Chemistry, Konkuk University, Chungju 27478, Republic of Korea; 3 Medical Proteomics Research Center, Korea Research Institute of Bioscience and Biotechnology, Daejeon 34141, Republic of Korea; University of Washington, UNITED STATES

## Abstract

Myotubularin-related protein 1 (MTMR1) is a phosphatase that belongs to the tyrosine/dual-specificity phosphatase superfamily. MTMR1 has been shown to use phosphatidylinositol 3-monophosphate (PI(3)P) and/or phosphatidylinositol 3,5-bisphosphate (PI(3,5)P_2_) as substrates. Here, we determined the crystal structure of human MTMR1. The refined model consists of the Pleckstrin homology (PH)-GRAM and phosphatase (PTP) domains. The overall structure was highly similar to the previously reported MTMR2 structure. Interestingly, two phosphate molecules were coordinated by strictly conserved residues located in the C(X)_5_R motif of the active site. Additionally, our biochemical studies confirmed the substrate specificity of MTMR1 for PI(3)P and PI(3,5)P_2_ over other phosphatidylinositol phosphates. Our structural and enzymatic analyses provide insight into the catalytic mechanism and biochemical properties of MTMR1.

## Introduction

Phosphatidylinositol polyphosphates (PIs; also called phosphoinositides) regulate diverse cellular functions, including membrane trafficking, cell growth/survival, cell division, and cellular motility [1]. The formation of seven different PIs is regulated by various kinases and phosphatases, including phosphatase and tensin homolog **(**PTEN), phosphatidylinositol-3-kinases (PI3Ks), phosphatidylinositol phosphate kinases (PIPKs), and myotubularin-related (MTMR) phospholipid phosphatases [1, 2]. Given the cellular functions of PIs, it is not surprising that PI dysregulation is associated with human diseases. Many mutations have been found in the kinases and phosphatases involved in PI interconversions [2, 3]. For example, mutations in inositol 5-phosphatase (OCRL) are associated with Lowe Syndrome and Dent Disease [4, 5], an inactivating mutation in PIP kinase type 1γ (PIPKIγ) is associated with Lethal Contractural Syndrome Type 3 (LCCS3) [6], and mutations in Fab 1, YOTB, Vac 1, and EEA (FYVE) finger-containing phosphoinositide kinase (PIKFyve) are associated with François-Neetens Mouchetée Fleck Corneal Dystrophy [7]. Moreover, inactivating mutations of PTEN and activating mutations of PI3Ks have been identified in various cancers because of their association with enhanced PI(3,4,5)P_3_ signaling [8–10]. Mutations in the myotubularin (MTM) family are also associated with human diseases. For instance, mutations in the MTM1 gene cause X-linked centronuclear myopathy and myotubular myopathy [11, 12]. Additionally, mutations in myotubularin-related protein 2 (MTMR2) and MTMR13 cause Charcot-Marie-Tooth Disease [13, 14].

MTMRs are part of a large group of tyrosine/dual-specificity phosphatase superfamily (PTP/DUSP) members in eukaryotes [15]. They are the primary enzymes involved in PI metabolism. The human MTMR family comprises 14 members. The eight MTMRs (MTM1, MTMR1–4, and MTMR6–8) that share the C(X)_5_R motif are catalytically active, and the other six MTMRs (MTMR5 and MTMR9–13), which do not possess the C(X)_5_R motif, are inactive phosphatases [16–18]. The inactive MTMRs interact with the active MTMRs and regulate the enzyme activity, subcellular localization, and substrate specificity of the active MTMRs [19–24]. Typically, the active MTMRs catalyze the dephosphorylation of phosphatidylinositol 3-monophosphate (PI(3)P) and/or phosphatidylinositol 3,5-bisphosphate (PI(3,5)P_2_) to produce PI and PI(5)P, respectively [1, 16–18]. Most MTMRs possess Pleckstrin homology (PH)-GRAM and coiled-coil (CC) domains in addition to the phosphatase (PTP) domain, and some MTMRs contain extra domains, such as differentially expressed in normal and neoplastic cells (DENN), PH, and FYVE domains [16]. The PH-GRAM domain has been predicted to function in intracellular protein-protein or lipid-binding interactions [1, 16, 17, 21, 25]. The CC domain mediates interactions between MTMRs and enables the homo- and/or heterodimerization of MTMRs [19, 21]. Human MTMR1, which is located on the X chromosome (similar to MTM1), was the first identified MTMR [26]. MTMR1 is associated with myotonic dystrophy and the efficient dephosphorylation of PI(3)P and PI(3,5)P_2_ *in vitro* [26–29]. Although MTMR1 was one of the earliest identified MTMRs, it is a relatively poorly studied protein. The crystal structures of MTMR2 [30], MTMR6 (Structural Genomics Consortium, unpublished work), and MTMR8 [31] provided detailed information regarding their substrate specificity and regulatory mechanisms based on their dimerization, protein-protein interactions and membrane binding. Thus, determining the 3-dimensional structure of MTMR1 would be indispensable for understanding the structural basis of its substrate specificity and regulatory mechanism. These biochemical data for MTMR1 could reveal the cellular mechanism underlying myotonic dystrophy.

In this study, we determined the crystal structure of the PH-GRAM and active PTP domains of human MTMR1 and investigated its substrate specificity through enzymatic and mutagenesis assays. We also determined the structural aspects of substrate recognition, the catalytic mechanism, and the regulatory mechanism of human MTMR1.

## Materials and Methods

### Materials

Di-C8 phosphatidylinositol 3-phosphate (di-C8 PI(3)P), di-C8 phosphatidylinositol 4-phosphate (di-C8 PI(4)P), di-C8 phosphatidylinositol 3,4-bisphosphate (di-C8 PI(3,4)P_2_), di-C8 phosphatidylinositol 3,5-bisphosphate (di-C8 PI(3,5)P_2_), di-C8 phosphatidylinositol 4,5-bisphosphate (di-C8 PI(4,5)P_2_), and di-C8 phosphatidylinositol 3,4,5-triphosphate (di-C8 PI(3,4,5)P_3_) were purchased from Cayman Chemical (USA). Malachite green reagent was prepared as previously described [32]. The crystallization screening reagents were purchased from Hampton Research (USA), Microlytic (USA), and Molecular Dimensions (UK).

### Purification, crystallization, and X-ray analysis

The cloning of the gene and expression, purification, and crystallization of the human MTMR1 protein have been described elsewhere [33]. Briefly, the MTMR1 gene (residues 95‒608, C438S mutant) was inserted into a modified pET32 vector (Novagen) using the NdeI/XhoI restriction enzyme sites. The resulting MTMR1 protein was fused with an octa-polyhistidine tag at its C terminus. The recombinant protein was overexpressed in *Escherichia coli* Rosetta 2(DE3) cells (Novagen). The protein was purified using Ni-NTA (Qiagen) and HiLoad 16/600 Superdex 200 prep-grade columns (GE Healthcare). The purified protein was concentrated to 13.5 mg/ml and stored in a buffer containing 20 mM Tris-HCl (pH 8.0), 300 mM NaCl, 10 mM Na_2_HPO_4_, and 5 mM dithiothreitol. The crystals were grown using the sitting drop vapor diffusion method. Each sitting drop was prepared by mixing 1 μl of purified protein and 1 μl of reservoir solution of 100 mM Na-HEPES (pH 7.0) and 15% (v/v) polyethylene glycol 20,000 in a 96-well crystallization plate. The reservoir solution supplemented with 25% (v/v) glycerol was used as a cryoprotectant solution. The X-ray diffraction data were collected using an ADSC Q315r detector at the Beamline 5C of the Pohang Light Source (Pohang, South Korea). The X-ray diffraction data were collected at 2.0 Å resolution. The data sets were processed and scaled using HKL2000 [34]. The detailed data and statistics are summarized in Table 1.

**Table 1:** Data collection and refinement statistics.

**Data collection**	
Diffraction source	PLS-5C
Wavelength (Å)	0.9796
Temperature (K)	100
Detector	ADSC Q315r
Space group	P1
Unit cell parameters (Å, °)	a = 67.219, b = 96.587, c = 97.581, α = 87.597, β = 86.072, γ = 77.327
Resolution range (Å)	50.0–2.07
Unique reflections	125,433
Redundancy	2.5 (2.2)^a^
Completeness (%)	86.1 (84.0)
Wilson B factor (Å^2^)	21.8
R_merge_ (%)^b^	6.4 (25.5)
I/sigma (I)	18.9 (3.2)
**Model refinement**	
Resolution (Å)	34.8–2.07
R_work_/R_free_ (%)^c^	22.0/27.4
Number of atoms (protein/water/phosphate)	15,824/652/30
Average B factor (protein/water/phosphate)	36.4/31.4/44.4
r.m.s. deviations (bond lengths (Å)/bond angles (°))	0.008/1.040
Ramachandran plot (favored/outliers (%))	96.03/0.10
PDB code	5C16

^a^The values in parentheses refer to the three highest-resolution shells (2.11–2.07).

^b^R_merge_ = Σ_h_Σ_i_|I(h)_i_–<I(h)>|/Σ_h_Σ_i_I(h)_i_, where I(h) is the intensity of reflection h, Σ_h_ is the sum over all reflections, and Σ_i_ is the sum over i measurements of reflection h.

^c^R_work_ = Σ | |F_obs_|–|F_calc_| | / Σ |F_obs_|, where R_free_ is calculated for a randomly chosen 5% of the reflections, which were not used for structure refinement, and R_work_ is calculated for the remaining reflections.

### Structure determination and homology modeling

The crystal structure of MTMR1 was solved by molecular replacement methods using the MOLREP program [35] in the CCP4 program package [36]. The human MTMR2 structure (PDB entry 1LW3) was used as the search model. The model was manually constructed using the Coot program [37]. The REFMAC 5 [38] and PHENIX [39] programs were used for model refinement. The final model for MTMR1 yielded R_factor_ and R_free_ values of 22.0 and 27.4%, respectively, with good stereochemistry. The refinement data were validated by the PROCHECK program [40]. The detailed statistics for refinement are also summarized in Table 1. Homology modeling for other MTMRs (MTMR3, 4, 5, 7, 8, 9, 10, 11, 12 and MTM1) was performed using the Schrödinger program package or SWISS-MODEL [41, 42].

### Immunoprecipitation

MTMR1 constructs including or lacking the CC domain (95‒665 or 95‒607) were cloned into the p3xFLAG-CMV-10 (Sigma-Aldrich) or pcDNA3-HA (Invitrogen) vectors, respectively. HEK293 cells were cotransfected with FLAG- and influenza hemagglutinin (HA)-tagged MTMR1 expression vectors using polyethylenimine (PEI; Sigma-Aldrich). The cells were harvested 40 hours after transfection. The immunoprecipitation experiment was performed using an anti-FLAG-M2 affinity gel (Sigma-Aldrich). The proteins were separated via 10% sodium dodecyl sulfate-polyacrylamide electrophoresis SDS-PAGE, and the western blot was performed with an anti-HA antibody (Cell Signaling Technology) and an anti-FLAG antibody (Sigma-Aldrich).

### Enzyme assay

For the malachite green assay with di-C8 PIs, 4 μL of 1 mM di-C8 PIs was added to 15 μL of assay buffer (50 mM Tris-HCl, pH 8.0, 10 mM dithiothreitol (DTT), and 0.5% NP-40). After prewarming at 37°C for 30 min, the reactions were initiated by the addition of ~2 μg of MTMR1. The reactions were quenched after 5 min by the addition of 20 μL of 0.1 M N-ethylmaleimide and centrifuged at 18,000 x g for 10 min. Then, 25 μL of the supernatant was added to 50 μL of the malachite green reagent and vortexed. The samples were incubated for 30–40 min for color development prior to measuring the absorbance at 620 nm.

## Results and Discussion

**Fig 1:**
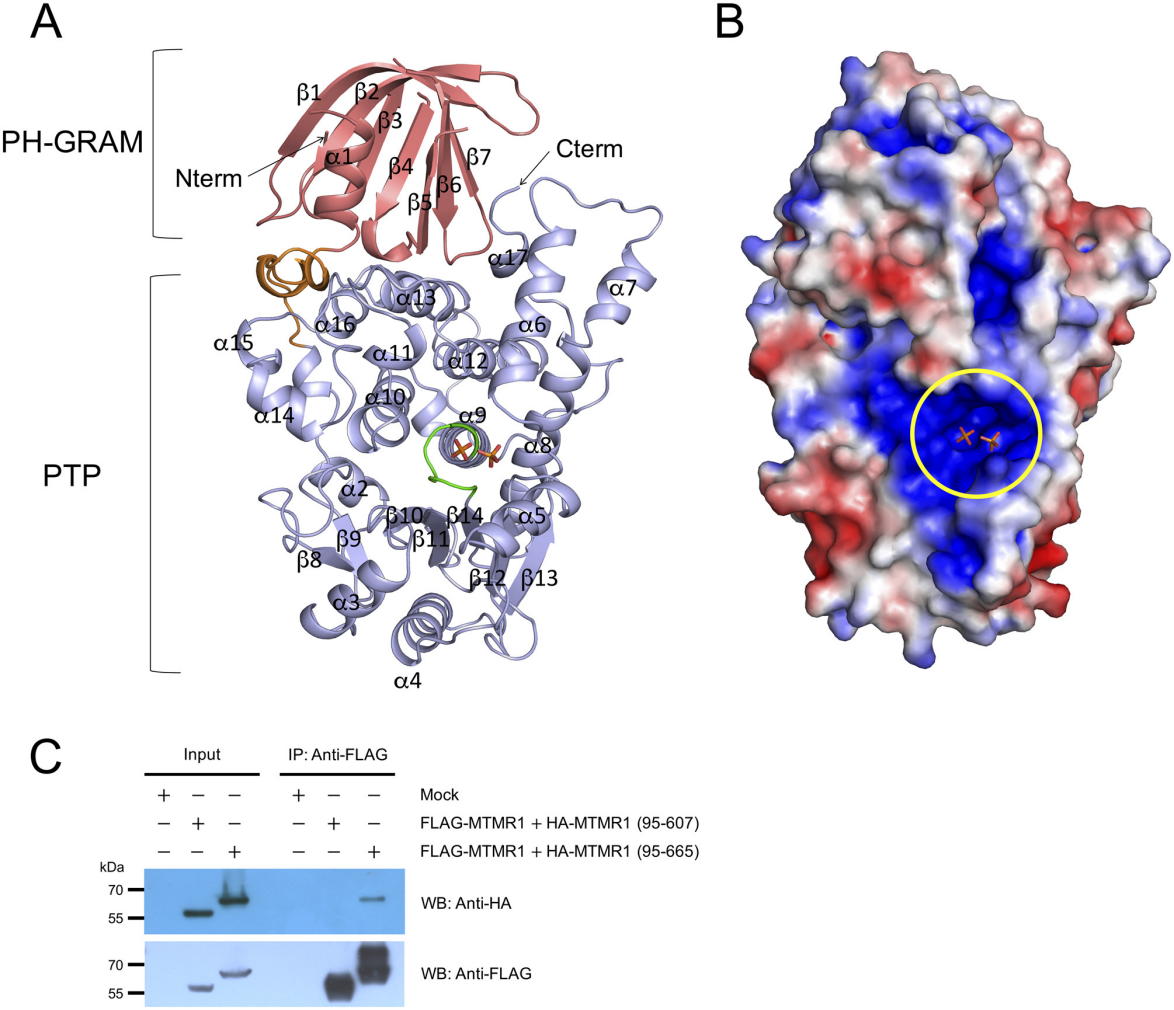
Overall structure of MTMR1. (A) A ribbon diagram of MTMR1. The PH-GRAM domain is depicted in pink, the PTP domain is in light blue, the linker is in orange, and the C(X)_5_R motif is in green. Two phosphate molecules are drawn. (B) The surface potential representation of MTMR1 with charge levels from -3kT/e (red) to +3kT/e (blue). The active site pocket containing the two phosphate molecules is indicated as a yellow circle. The surface potential representation was generated using PDB2PQR and APBS [48, 49]. The figures were drawn using PyMol (Schrödinger, LLC**)**. (C) Immunoprecipitation and western blot analysis with FLAG- and HA-tagged MTMR1. A strong interaction was observed between FLAG-MTMR1 and HA-MTMR1 (95–665), suggesting the formation of dimers, but no interaction was found with CC domain-truncated MTMR1 (95–607) constructs.

**Fig 2:**
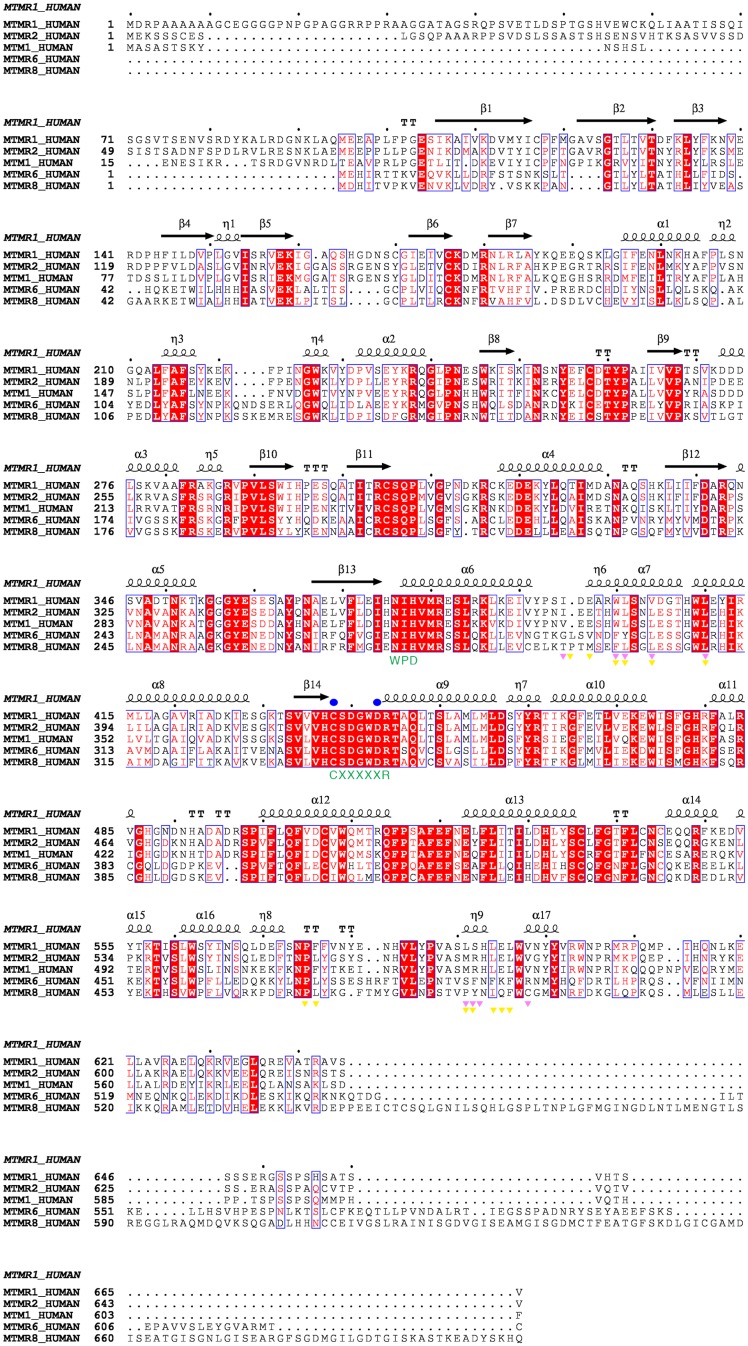
Alignments of the amino acid sequences of the MTMRs. The C(X)_5_R motif and residues corresponding to the WPD motif of the PTPs are indicated in green letters. The catalytically important residues are marked in blue circles. Residues involved in the dimeric interfaces for MTMR2 and MTMR8 are marked with pink (MTMR2) and yellow (MTMR8) triangles, respectively. This figure was drawn using Espript (http://espript.ibcp.fr) [50].

The MTMR1 structure was determined by molecular replacement using the MTMR2 structure as the search model and refined to 2.07 Å resolution. The 97 flexible N-terminal residues (residues 1–97) and the C-terminal CC domain region (residues 606–665) were excluded during gene cloning and were not modeled. The refined model included four MTMR1 molecules (A, B, C, and D chains), 652 water molecules, and 6 phosphate ions in the asymmetric unit. The overall conformations of the four molecules in the asymmetric unit were similar (S1 Fig). Three loops (140–143, 161–170 and 187–193) were not modeled because of their invisible electron density maps. The MTMR1 structure was refined to a crystallographic R-factor of 22.0% (R_free_ = 27.4%). The refined MTMR1 monomer structure was composed of 14 β-strands (β1−β14) and 17 α-helices (α1−α17) that formed two individual domains: the PH-GRAM domain (residues 98–205) and the PTP domain (residues 226–605) (Fig 1A). These two domains were connected by a linker that contained two short 3_10_-helices (residues 206–225). The PH-GRAM domain, which was originally implicated in protein or lipid binding during membrane-associated processes, exhibited a β-sandwich structure formed by seven β-strands (β1−β7) and one α-helix (α1) that was similar to the MTMR2 structure [25, 30]. The second domain of MTMR1 was a large PTP domain composed of seven β-strands (β8−β14) and sixteen α-helices (α2−α17). The central twisted β-sheet was flanked by several α-helices forming a large globular domain. The MTMR PTP domain was larger than other PTP domains, including those in PTEN, MKPs, and VHR [30, 43]. Similar to other members of the MTMR protein family, the PTP domain of MTMR1 contained a SET-Interaction Domain (SID; α12−α14) that might facilitate protein-protein interactions [30]. The residues of the C(X)_5_R motif in the active site (Cys438 (here, mutated to Ser438 for crystallization), Ser439, Asp440, Gly441, Trp442, Asp443, and Arg444) were aligned in a loop between β14 and α9 (Figs 1 and 2). The electrostatic surface potential showed a highly positive patch around the active site (Fig 1).

The molecular packing of MTMR1 in the crystal did not show any oligomeric association. The results of a PISA analysis, used to estimate the oligomeric states, did not indicate an oligomeric association [44]. The largest calculated interface area located between chains A and D or chains B and C was approximately 950 Å^2^ and had a zero Complex Formation Significance Score (CSS). In this study, the purified recombinant MTMR1 protein existed as a monomer based on the dynamic light scattering estimation (data not shown). However, MTMRs usually exist as dimers in solution [45]. This discrepancy suggested that the CC domain of MTMR1 might be essential for its dimerization. Notably, the association of MTMR2 with MTMR5 or itself through the CC domain is necessary for its enzymatic activity and cellular localization [19]. This result indicates the importance of the CC domain for the homo- or heterodimerization of MTMRs. Immunoprecipation experiments using FLAG- and HA-tagged MTMR1 with or without the CC domain showed that only CC domain-containing MTMR1 could exist as a dimer, suggesting the importance of the CC domain in the dimerization of MTMR1 (Fig 1). However, in MTMR8, only the PTP domain can exist as a dimer in solution, and its crystal structure corresponds to that of the MTMR8 dimer without the CC domain [31]. The dimer interfaces in MTMR8 are usually formed by hydrophobic residues in the α6−α7 loop, in α7, and in the α16−α17 loop [31]. However, the corresponding residues are less conserved in MTMR1, which may increase the contribution of the CC domain to the dimerization of MTMR1 compared with MTMR8 (Fig 2).

**Fig 3:**
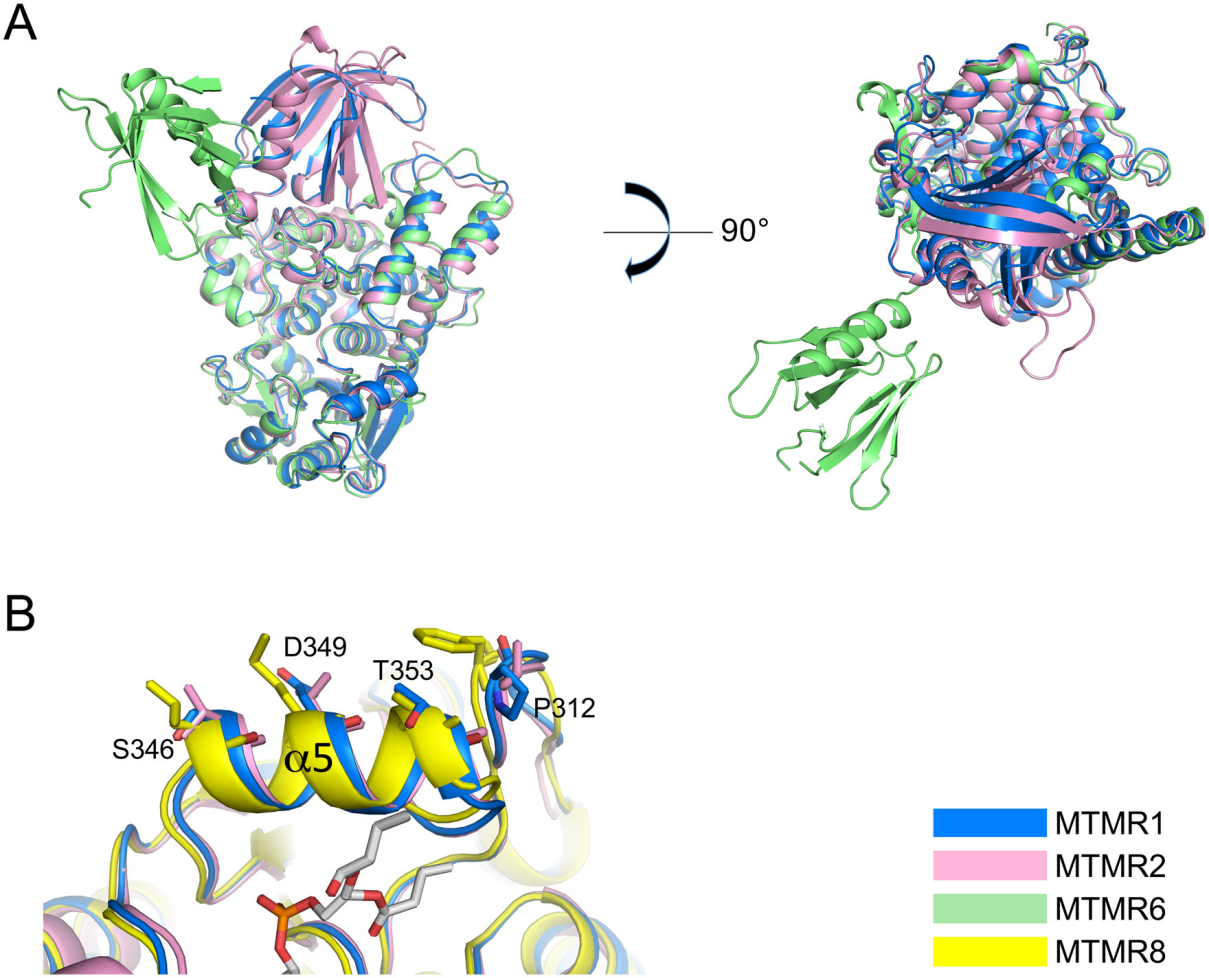
Comparison of the MTMR1, MTMR2, MTMR6, and MTMR8 structures. (A) Overall comparison of the MTMR1, MTMR2, and MTMR6 structures that contain the PH-GRAM and PTP domains. The sequence identities to MTMR1 are 67.6% for 678 MTMR2 residues, 36.9% for 694 MTMR6 residues, and 39.7% for 638 MTMR8 residues. (B) Comparison of the structures of the α5 helix, which is thought to be important for membrane association. Each MTMR is colored aqua, pink, lime, or yellow. The chain A molecule of each MTMR was used for the structural comparisons.

We compared the structure of human MTMR1 with the structures of MTMR2 (PDB code 1LW3 or 1ZVR), MTMR6 (PDB code 2YF0), and MTMR8 (PDB code 4Y7I). The MTMR1, MTMR2, and MTMR6 structures contain both the PH-GRAM and PTP domains, whereas the MTMR8 structure contains only the PTP domain [30, 31, 46]. MTMR1, MTMR2, MTMR6, and MTMR8 have a high degree of amino acid sequence conservation, and all of these MTMRs are catalytically active phosphatases [1] (Fig 2). The MTMR2 structure was easily superimposed with MTMR1, and its root-mean-square (r.m.s.) deviation was only 0.9 Å for the 513 Cα atom pairs comprising the PH-GRAM and PTP domains. In the MTMR1 and MTMR2 structures, the PH-GRAM domain bound tightly to the large and shallow cleft of the PTP domain formed by residues in and around three helices (α6, α13 and α17) and a loop located between α11 and α12 (Figs 1 and 3A, S1 Table). The MTMR6 structure exhibited a large conformational change (Fig 3A). The MTMR6 PH-GRAM domain was detached from the shallow cleft of the PTP domain, and the crystallographic symmetry operation revealed a domain-swapped dimeric formation of MTMR6 (S2 Fig). The domain-swapped PH-GRAM domain interacted with the PTP domain of an adjacent subunit. However, the relative orientation of these two domains of MTMR6 differed markedly from the MTMR1 and MTMR2 structures [31]. Specifically, the residues involved in the interactions between these two domains were less conserved in MTMR6. Only three residues out of 21 interface residues in the PH-GRAM domain and seven residues out of 27 residues in the PTP domain in MTMR6 were conserved (Fig 2 and S1 Table). Although a large conformational change was observed in MTMR6, the overall fold of the individual domains of MTMR6 was similar to that of MTMR1 or MTMR2. The PH-GRAM and CC domains of MTMR6 bind to another component in the plasma membrane and inhibit the Ca^2+^-activated K^+^ channel KCa3.1. Interestingly, other MTMRs cannot inhibit KCa3.1 [47]. This difference may be due to the different orientation of the MTMR6 domains compared with other MTMRs.

**Fig 4:**
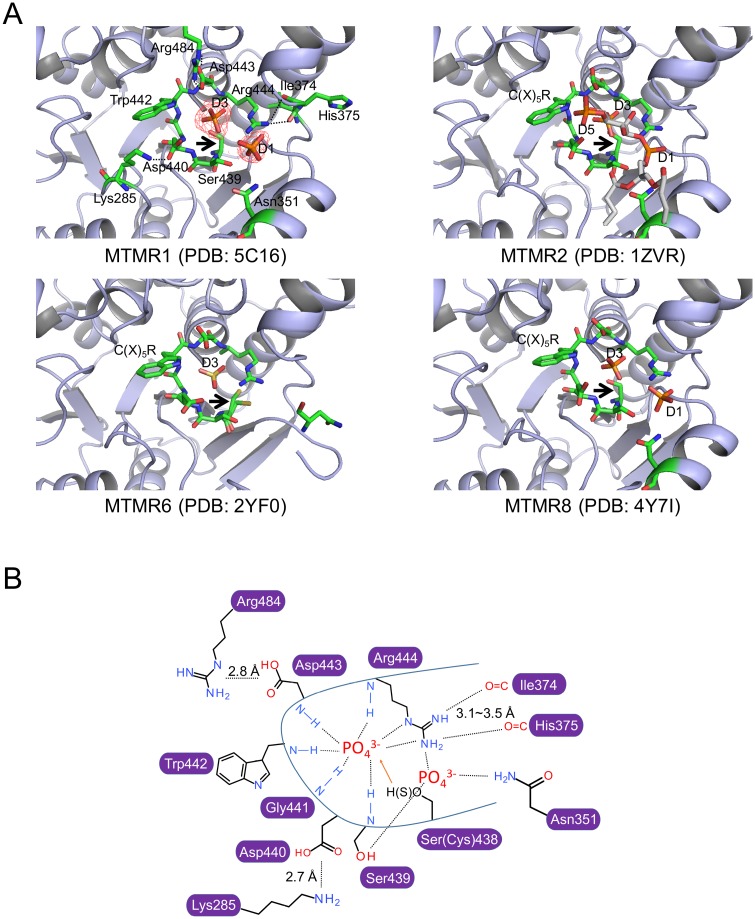
Active sites of the known MTMR structures. (A) Active sites of individual MTMRs. The residues on the C(X)_5_R loop and other important residues are shown using green stick models. The phosphates (in MTMR1 and MTMR8), sulfate (in MTMR6), or PI(3,5)P_2_ (in MTMR2) molecules are also depicted as stick models. The electron density map for the phosphate molecules in MTMR1 is shown with a red mesh. Black arrows point to the cysteine residue in the C(X)_5_R motif of the MTMRs that is mutated to serine in the MTMR structures, except for MTMR6, for crystallization purposes. (B) Illustrated representation of the phosphate-bound active site in the MTMR1 structure. The hydrogen bond distances between residues in the C(X)_5_R motif and the phosphate ions were approximately 2.5–3.0 Å.

In our structure, one or two phosphate molecules were observed in the active site pocket of each MTMR1 molecule. The phosphate molecules were clearly visible in the electron density map of the active site (Fig 4A). These two phosphates correspond nicely with the D1 and D3 sites of PI(3,5)P_2_ bound to MTMR2 (PDB code, 1ZVR). Among them, one phosphate molecule (D3 phosphate site of PI) was positioned in the deep cleft of the active site pocket and was mainly trapped by its interaction with the main chains of the six residues (Ser(Cys)438, Ser439, Gly441, Trp442, Asp443, and Arg444) from the C(X)_5_R motif. Only Asp440 in the C(X)_5_R motif did not interact with this phosphate. This D3 phosphate ion is observed in all four MTMR1 chains in the asymmetric unit. The side chains of Asp440 and Asp443 are positioned away from the active site cavity because of the formation of hydrogen bonds with the side chains of Lys285 and Arg484, respectively, creating sufficient space for the phosphate ion or D3 portion of the substrate (Fig 4B). The side chain conformation of Arg444 is restrained by the main chains of Ile374 and His375, enabling this residue to interact with the phosphates. The second phosphate molecule, which is positioned at the D1 site of PI and only found in two MTMR1 molecules (chains A and B) in the asymmetric unit, interacts with the side chains of Asn351, Ser439, and Arg444. Asn351 lies on helix α5 and is strictly conserved across the MTMRs (Fig 2), suggesting its importance in binding PI. Interestingly, in contrast to the other MTMR structures, the MTMR6 structure contains only one sulfate ion in the active site at the D3 position (Fig 4). Although the amino acid sequence is still highly conserved, the helix corresponding to α5 in MTMR1 is missing in the MTMR6 structure, indicating its flexibility. It is unclear whether the missing structure of this helix is the result of the crystal packing or another reason. Additionally, it should be noted that α5 has been thought to be important for membrane association in MTMR2 and MTMR8 [31]. The well-conserved hydrophobic residues (Val325, V328, and Ala332 in MTMR2, and Leu245, Met248, and Ala252 in MTMR8) are exposed to the surface, facilitating membrane association (Fig 3). However, in MTMR1, the corresponding residues are usually hydrophilic (Ser346, Asp349, and Thr350), suggesting a different mode of membrane association (Fig 3). The phosphate binding in the active site could provide insight into the substrate-binding mode and enable us to construct a model for PI-bound MTMR1 (S3 Fig). Many MTMR structures do not contain any phosphate ions at the D5 phosphate site of PI. The MTMR2 structure in complex with PI(3,5)P_2_ shows that the D5 phosphate site is solvent-exposed and has fewer contacts than the other phosphate sites [46], which may explain the absence of the phosphate ion at the D5 phosphate position in the active site. Therefore, it is not surprising that MTMRs dephosphorylate the D3 phosphate from PI(3)P or PI(3,5)P_2_ and do not recognize the D5 phosphate of the substrates. Unlike PTEN, MTMRs cannot dephosphorylate PIs that are phosphorylated at the D4 position. This difference may be due to the presence of bulky tryptophan and arginine residues (Trp442 and Arg484 in MTMR1) in the C(X)_5_R motif, as previously discussed [46].

Generally, the C(X)_5_R motif loop in the active site builds the framework of the substrate-binding pocket and determines the substrate specificity depending on its depth [16]. Similar to MTMR2, the membrane-proximal surface of the active site of MTMR1 is highly positive (Fig 1B) and is relatively wider and deeper than the sites of phospho-Ser/Thr/Tyr-specific PTPs, allowing it to accommodate relatively bulky substrates, such as PIs [16]. The cysteine residue (Cys438 in MTMR1) in this motif attacks the phosphorous atom of the substrate as a nucleophile, generating a phosphoenzyme intermediate and an aspartic acid (Asp443 in MTMR1) that acts as both a general acid and base for the dephosphorylation reaction [16, 18, 46]. In our structure, the side chain of Asp443, which is tethered by the Arg484 side chain, is positioned within possible hydrogen bonding distance of a phosphate ion (Fig 4). Canonical PTPs position their general acid/base (usually an aspartate) on the WPD-loop of the PTP domain. However, in MTMRs, this aspartate residue lies on the C(X)_5_R instead of the WPD-loop (Fig 2). This feature is one that distinguishes MTMRs from other PTPs. When we constructed homology models of MTM1 and all of the MTMRs for which crystal structures had not been determined, all of the models included the PH-GRAM and PTP domains (S4 Fig). Moreover, although most of the catalytically active MTMRs show positive surface charges around the active sites, all of the catalytically inactive MTMRs (with the exception of MTMR9) have a negative patch in the corresponding region. However, the inactive MTMRs possess relatively large positive patches on their dead active site faces, thereby facilitating membrane binding.

**Fig 5:**
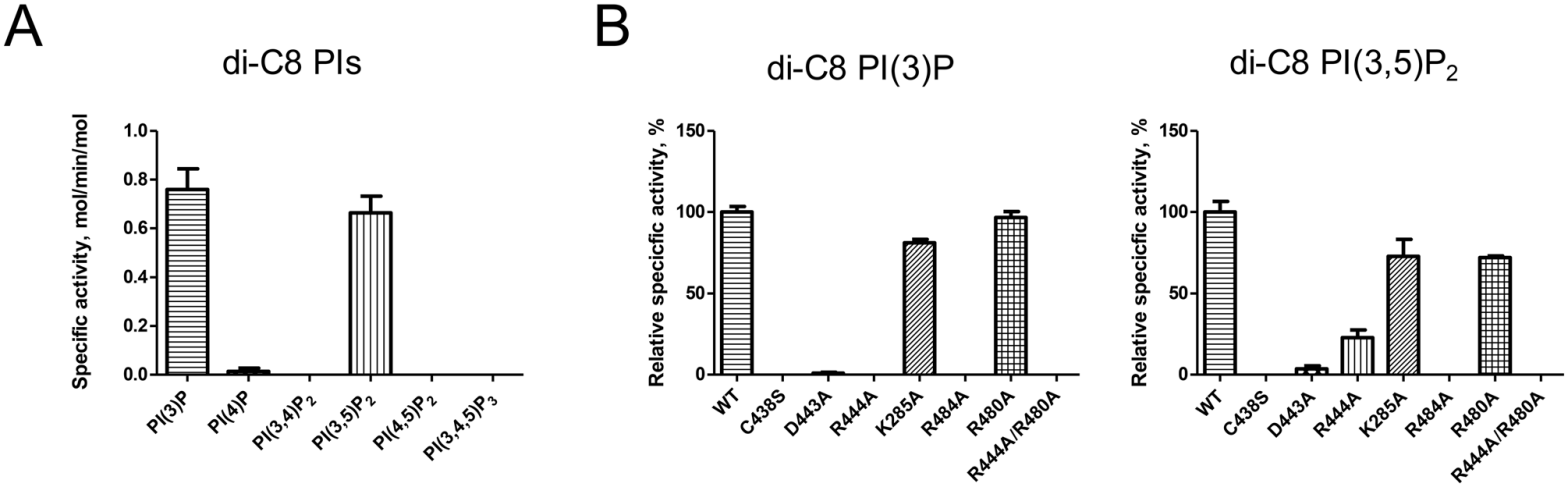
Enzymatic activity of MTMR1. (A) Specific activity of MTMR1 against di-C8 PIs. (B) Comparison of the specific activities of wild-type and mutant (C438S, D443A, R444A, K285A, R484A, R480A, and R444A/R480A) MTMR1 against di-C8 PI(3)P and di-C8 PI(3,5)P_2_. The reactions were performed in triplicate, and the specific activities are presented as moles of phosphate released per minute per mole of enzyme ± SD.

Next, we tested the substrate specificity of MTMR1 toward di-C8 PIs. MTMR2 exhibits phosphatase activity against both PI(3)P and PI(3,5)P_2_ [29, 30]. However, previous studies have reported different results regarding its substrate specificity against PI(3)P and PI(3,5)P_2_ [28]. In this study, the purified recombinant MTMR1 exhibited phosphatase activities against both di-C8 PI(3)P and di-C8 PI(3,5)P_2_ (Fig 5A). We also tested whether the active site residues affected the phosphatase activity. As expected, three MTMR1 mutants (C438S, D443A, and R444A) showed no (or reduced) phosphatase activity. As explained previously, Cys438 functions as a nucleophile, and Asp443 is a general acid and base. Therefore, these two residues are essential for the phosphatase activity. In the MTMR1 structure, Arg444 plays an important role in binding to the phosphate group of the substrate, which explains the reduced phosphatase activity of the mutant. Then, we produced additional MTMR1 mutants and checked their enzymatic activities (K285A, K484A, R480A, and R444A/R480A). Here, K285 is a hydrogen-bonding partner with Asp440, and Arg484 interacts with Asp443 (Fig 4). R480 corresponds to R459 in MTMR2, which interacts with the D5 phosphate of PI(3,5)P_2_. The mutation of Lys285 had little effect on the phosphatase activity, whereas the R484A mutant exhibited no phosphatase activity. Arg484 may play critical roles in contributing to the structural restraints in the C(X)5R loop of MTMR1 in its enzymatic reaction. As expected, although the R480A mutation did not affect phosphatase activity with PI(3)P, a ~ 25% reduction in phosphatase activity was observed for PI(3,5)P_2_. Interestingly, a partial recovery of the specific activity was observed in the reaction of the R444A mutant with PI(3,5)P_2_ because of the contribution of the D5 phosphate instead of the D3 phosphate. The R444A/R480A double mutant did not show any enzymatic activity toward either PI(3)P or PI(3,5)P_2_.

In summary, we determined the crystal structure of human MTMR1. The MTMR1 structure contained two phosphate ions in the active site pocket that could promote substrate binding. Moreover, the phosphatase assay showed high substrate selectivity for both PI(3)P and PI(3,5)P_2_, similar to MTMR2.

## Supporting Information

S1 Fig Structural superposition of four molecules in the asymmetric unit of the MTMR1 crystal. (A) Stereo view of the superposition of each MTMR1 molecule in the asymmetric unit. (B) Stereo view of the active site using four superposed MTMR1 molecules in the asymmetric unit (C) Calculated r.m.s.d. values between each MTMR1 molecule. Chain A, Chain B, Chain C, and Chain D are shown in green, cyan, magenta, and yellow, respectively.
(PDF)

S2 Fig Domain-swapped structure of MTMR6 (PDB code 2YF0). Each subunit was drawn in red or cyan.
(PDF)

S3 Fig Modeled structure of PI(3,5)P_2_-bound MTMR1. The structure of MTMR2 in complex with PI(3,5)P_2_ (PDB code, 1ZVR) was used for modeling. The electron density map of two phosphates in MTMR1 is shown.
(PDF)

S4 Fig Comparison of the structures of MTM1 and 13 MTMRs. Electrostatic surface potential of MTM1 and the MTMRs. All structures include the PH-GRAM and PTP domains. MTMR1, MTMR2, and MTMR6 are crystal structures. MTMR8 is a partially modeled structure. The other MTMRs and MTM1 are homology-modeled structures. All structures are drawn in the same orientation, and the yellow arrows indicate the active site (C(X)_5_R loop) for each PTP domain. Active MTMRs are indicated in red, and inactive MTMRs are indicated in green. The MTMR6 structure was used to model MTMR7 and MTMR8, and the MTMR2 structure was used to model the other MTMRs. The modeled structures for MTMR7 and MTMR8, which have relatively high amino acid sequence similarities to MTMR6, adopt conformations similar to that of MTMR6. However, no experimental evidence indicates that these proteins will adopt such conformations.
(PDF)

S1 Table Interface residues involved in the PH-GRAM and PTP interactions in MTMR1. (PDF)
