# Correction: Crystal Structure of Human Myotubularin-Related Protein 1 Provides Insight into the Structural Basis of Substrate Specificity

**DOI:** 10.1371/journal.pone.0190844

**Published:** 2018-01-02

**Authors:** Seoung Min Bong, Kka-bi Son, Seung-Won Yang, Jae-Won Park, Jea-Won Cho, Kyung-Tae Kim, Hackyoung Kim, Seung Jun Kim, Young Jun Kim, Byung Il Lee

There is an error in the second sentence of the “Purification, crystallization, and X-ray analysis” section of the Materials and Methods. The correct sentence is: Briefly, the MTMR1 gene (residues 95‒608, C438S mutant) was inserted into a modified pET28 vector (Novagen) using the NdeI/XhoI restriction enzyme sites.

## References

[1] Bong SM, Son K-b, Yang S-W, Park J-W, Cho J-W, Kim K-T, et al (2016) Crystal Structure of Human Myotubularin-Related Protein 1 Provides Insight into the Structural Basis of Substrate Specificity. PLoS ONE 11(3): e0152611 https://doi.org/10.1371/journal.pone.0152611 27018598 10.1371/journal.pone.0152611 PMC4809516

