# Supplementary material for: Crystal Structure of Human Myotubularin-Related Protein 1 Provides Insight into the Structural Basis of Substrate Specificity
Source: PLoS One. 2016 Mar 28;11(3):e0152611. doi: 10.1371/journal.pone.0152611 (PMC4809516; doi:10.1371/journal.pone.0152611)

S1 Figure. Structural superposition of four molecules in the asymmetric unit of the MTMR1 crystal. (A) Stereo view of the superposition of each MTMR1 molecule in the asymmetric unit. (B) Stereo view of the active site using four superposed MTMR1 molecules in the asymmetric unit (C) Calculated r.m.s.d. values between each MTMR1 molecule. Chain A, Chain B, Chain C, and Chain D are shown in green, cyan, magenta, and yellow, respectively.

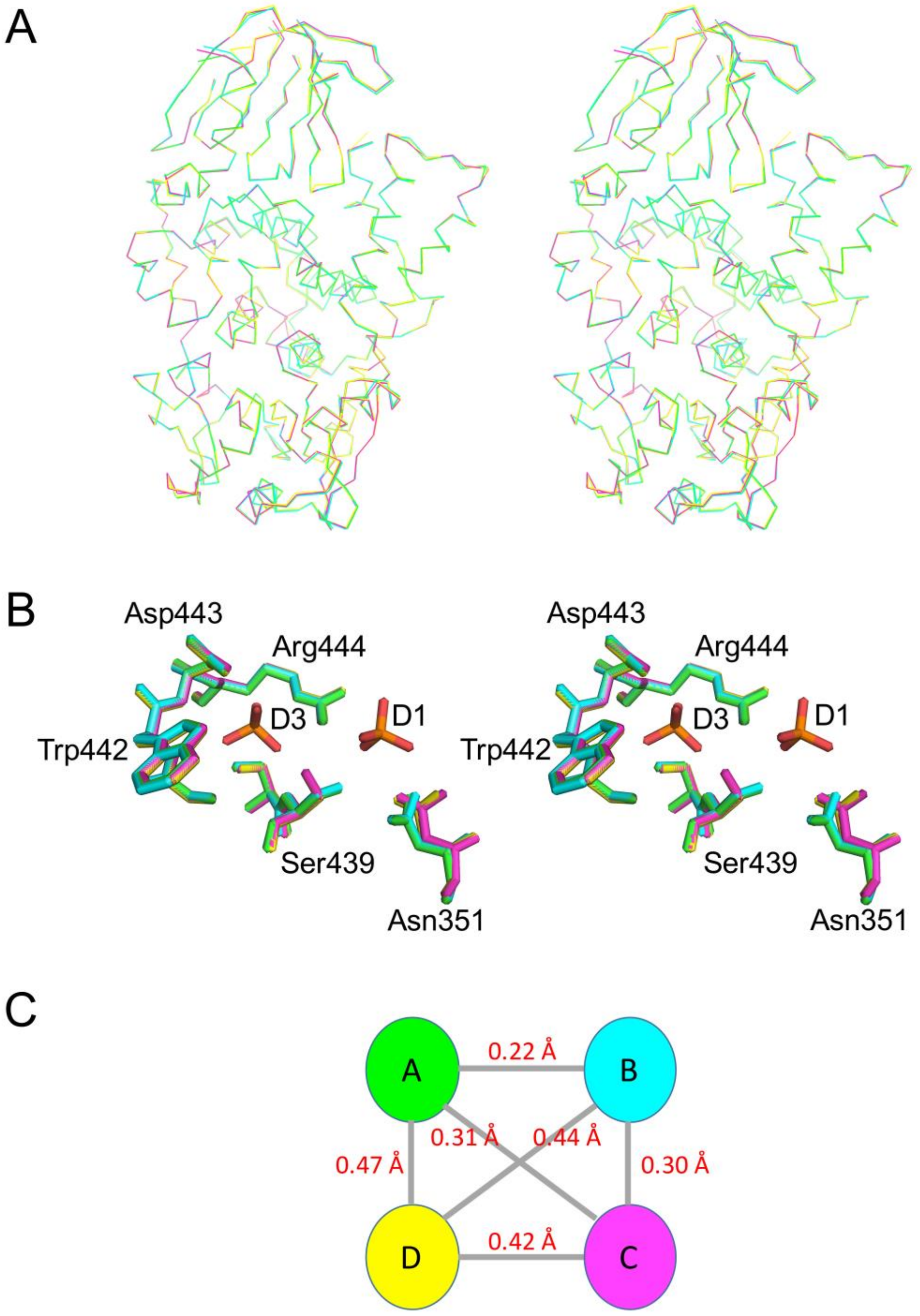

Supplement: S1 Fig — (A) Stereo view of the superposition of each MTMR1 molecule in the asymmetric unit. (B) Stereo view of the active site using four superposed MTMR1 molecules in the asymmetric unit (C) Calculated r.m.s.d. values between each MTMR1 molecule. Chain A, Chain B, Chain C, and Chain D are shown in green, cyan, magenta, and yellow, respectively. (PDF) [file pone.0152611.s001.pdf]
