# Supplementary figures and images for: Crystal Structure of Human Myotubularin-Related Protein 1 Provides Insight into the Structural Basis of Substrate Specificity
Source: PLoS One. 2016 Mar 28;11(3):e0152611. doi: 10.1371/journal.pone.0152611 (PMC4809516; doi:10.1371/journal.pone.0152611)

**S2 Figure.** Domain-swapped structure of MTMR6 (PDB code 2YF0). Each subunit was drawn in red or cyan.

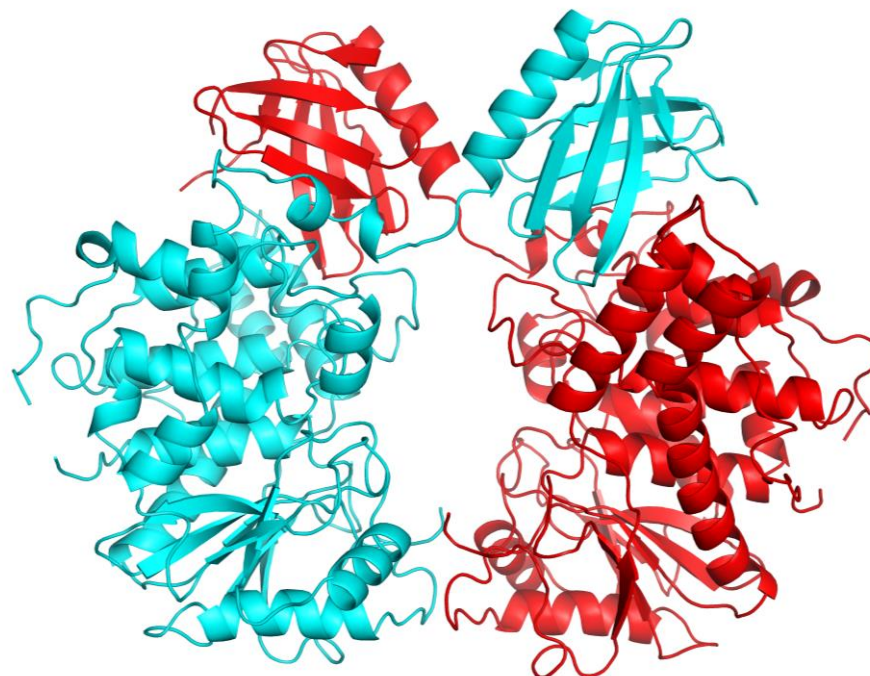

Supplement: S2 Fig — Each subunit was drawn in red or cyan. (PDF) [file pone.0152611.s002.pdf]
