# Supplementary material for: Crystal Structure of Human Myotubularin-Related Protein 1 Provides Insight into the Structural Basis of Substrate Specificity
Source: PLoS One. 2016 Mar 28;11(3):e0152611. doi: 10.1371/journal.pone.0152611 (PMC4809516; doi:10.1371/journal.pone.0152611)

S3 Figure. Modeled structure of PI(3,5)P_2-bound MTMR1. The structure of MTMR2 in complex with PI(3,5)P_2 (PDB code, 1ZVR) was used for modeling. The electron density map of two phosphates in MTMR1 is shown.

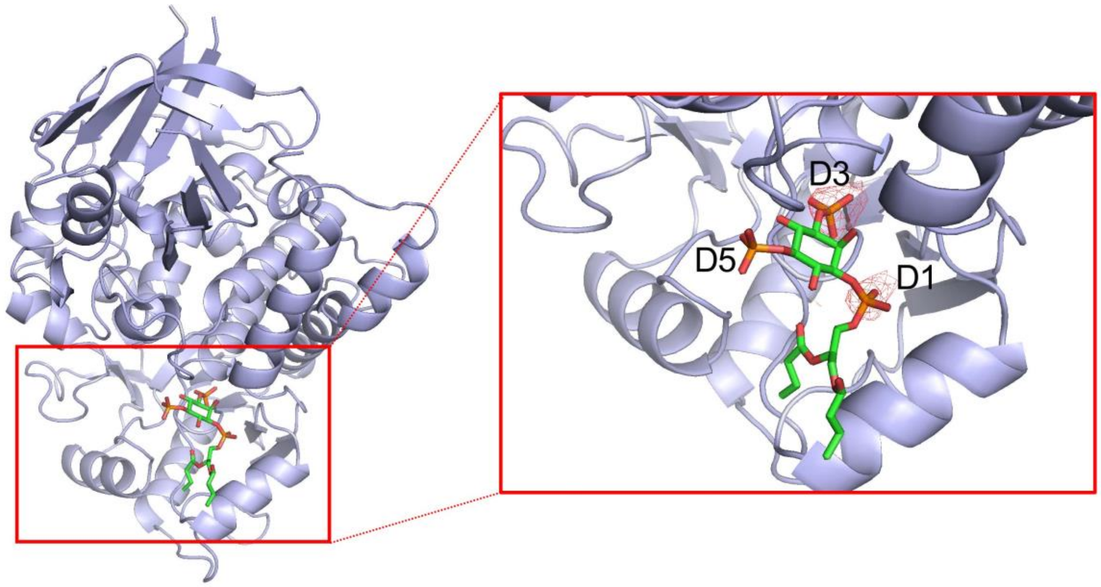

Supplement: S3 Fig — The structure of MTMR2 in complex with PI(3,5)P2 (PDB code, 1ZVR) was used for modeling. The electron density map of two phosphates in MTMR1 is shown. (PDF) [file pone.0152611.s003.pdf]
