# Supplementary material for: Crystal Structure of Human Myotubularin-Related Protein 1 Provides Insight into the Structural Basis of Substrate Specificity
Source: PLoS One. 2016 Mar 28;11(3):e0152611. doi: 10.1371/journal.pone.0152611 (PMC4809516; doi:10.1371/journal.pone.0152611)

S4 Figure. Comparison of the structures of MTM1 and 13 MTMRs. Electrostatic surface potential of MTM1 and the MTMRs. All structures include the PH-GRAM and PTP domains. MTMR1, MTMR2, and MTMR6 are crystal structures. MTMR8 is a partially modeled structure. The other MTMRs and MTM1 are homology-modeled structures. All structures are drawn in the same orientation, and the yellow arrows indicate the active site ($C(X)_5R$ loop) for each PTP domain. Active MTMRs are indicated in red, and inactive MTMRs are indicated in green. The MTMR6 structure was used to model MTMR7 and MTMR8, and the MTMR2 structure was used to model the other MTMRs. The modeled structures for MTMR7 and MTMR8, which have relatively high amino acid sequence similarities to MTMR6, adopt conformations similar to that of MTMR6. However, no experimental evidence indicates that these proteins will adopt such conformations.

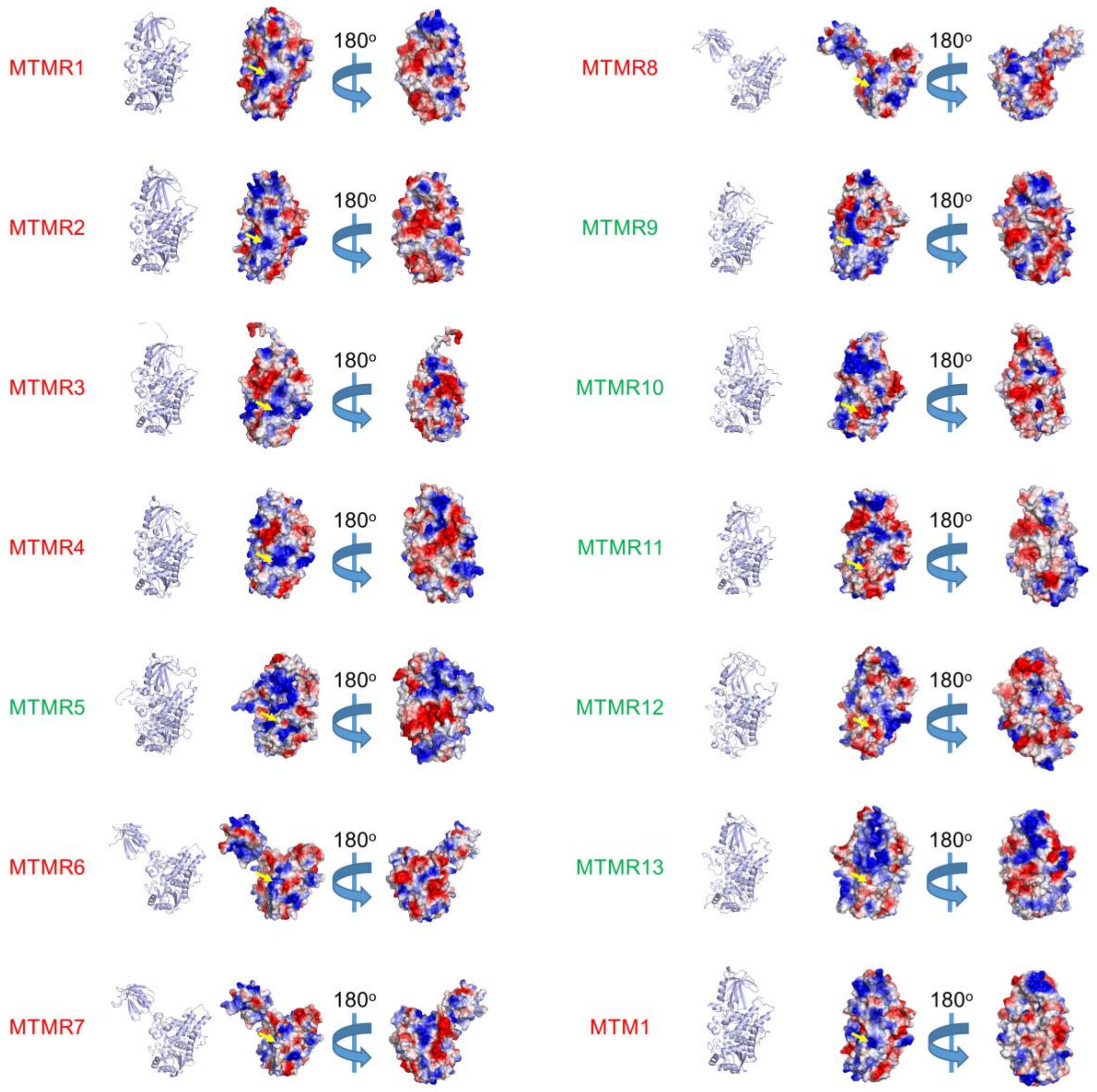

Supplement: S4 Fig — Electrostatic surface potential of MTM1 and the MTMRs. All structures include the PH-GRAM and PTP domains. MTMR1, MTMR2, and MTMR6 are crystal structures. MTMR8 is a partially modeled structure. The other MTMRs and MTM1 are homology-modeled structures. All structures are drawn in the same orientation, and the yellow arrows indicate the active site (C(X)5R loop) for each PTP domain. Active MTMRs are indicated in red, and inactive MTMRs are indicated in green. The MTMR6 structure was used to model MTMR7 and MTMR8, and the MTMR2 structure was used to model the other MTMRs. The modeled structures for MTMR7 and MTMR8, which have relatively high amino acid sequence similarities to MTMR6, adopt conformations similar to that of MTMR6. However, no experimental evidence indicates that these proteins will adopt such conformations. (PDF) [file pone.0152611.s004.pdf]
