# Supplementary material for: Crystal Structure of Human Myotubularin-Related Protein 1 Provides Insight into the Structural Basis of Substrate Specificity
Source: PLoS One. 2016 Mar 28;11(3):e0152611. doi: 10.1371/journal.pone.0152611 (PMC4809516; doi:10.1371/journal.pone.0152611)
Supplement: S1 Table — (PDF) [file pone.0152611.s005.pdf]

S1 Table. Interface residues involved in the PH-GRAM and PTP interactions in MTMR1.

PH-GRAM					PTP				
Residue[a]	HSDC[b]	ASA[c]	BSA[d]	Δ^iG[e]	Residue	HSDC	ASA	BSA	Δ^iG
Phe101 (Thr)		122.17	29.83	0.48	Leu384 (Leu)		38.54	17.73	0.28
Tyr115 (Ser)		22.78	3.66	-0.04	Leu387 (Leu)		1.31	1.31	0.02
Pro118 (Ser)		118.28	59.43	0.95	Lys388 (Leu)	H	136.53	98.90	-0.92
Phe119 (Leu)		173.19	55.88	0.89	Val391 (Asn)		13.96	8.17	0.13
Phe132 (Thr)		86.29	52.71	0.84	Tyr392 (Gly)	H	119.35	88.54	0.42
Pro150 (Leu)		40.48	14.23	0.23	Pro393 (Thr)		91.62	30.40	0.44
Gly152 (His)		54.90	38.82	0.48	Ser394 (Lys)		80.04	28.86	0.37
Val153 (His)		30.13	25.61	0.41	Val485 (Cys)	H	31.64	17.13	-0.08
Ser155 (Ala)		52.46	34.20	-0.24	Gly486 (Gly)		11.98	6.00	-0.02
Arg156 (Ser)		124.32	46.19	-0.31	His487 (Gln)		37.08	3.33	0.38
Val175 (Gln)		45.75	6.68	0.11	Gly488 (Leu)		93.17	36.08	0.07
Cys176 (Cys)	H	12.43	8.63	-0.10	Asn489 (Asp)		87.72	10.72	-0.05
Lys177 (Lys)	H	138.45	103.07	-1.03	Asp490 (Gly)		137.13	52.04	0.51
Asp178 (Asn)		60.86	57.73	0.24	His492 (Pro)		155.67	82.72	0.35
Met179 (Phe)		185.10	178.90	3.01	Arg497 (-)		45.31	19.66	-0.73
Arg180 (Arg)	H	87.55	73.99	-0.22	Leu502 (Thr)		3.62	1.84	0.03
Asn181 (Thr)	H	85.44	46.99	-0.31	Asp531 (Glu)		52.15	12.10	-0.09
Leu182 (Val)		5.82	0.17	0.00	His532 (His)		33.86	1.88	0.03
Asn201 (Leu)		56.71	9.89	-0.11	Tyr534 (His)		110.80	94.72	0.92
Ala204 (Ser)		15.39	8.62	-0.09	Ser535 (Ser)		62.33	57.20	0.40
Phe205 (Lys)		133.14	64.52	0.96	Cys536 (Cys)		54.92	4.95	0.13
					Leu537 (Gln)		119.21	52.71	0.84
					Tyr600 (Met)		29.21	28.49	0.03
					Tyr601 (Tyr)		21.89	20.54	0.04
					Arg603 (Gln)		74.88	6.41	-0.07
					Trp604 (Phe)		180.98	90.25	0.90
					Asn605 (Asp)		152.18	32.61	-0.37

[a]Amino acids in the parentheses are corresponding residues in MTMR6. Strictly conserved residues are colored in red.
[b]HSDC, Residues making Hydrogen/Disulphide bond, Salt bridge or Covalent link.
[c]ASA, Accessible Surface Area (Å ^{2}).
[d]BSA, Buried Surface Area (Å ^{2}).
[e]Δ^iG, Solvation energy effect (kcal/mol).
